# Compromised MPS1 Activity Induces Multipolar Spindle Formation in Oocytes From Aged Mares: Establishing the Horse as a Natural Animal Model to Study Age-Induced Oocyte Meiotic Spindle Instability

**DOI:** 10.3389/fcell.2021.657366

**Published:** 2021-05-06

**Authors:** Marilena Rizzo, Tom A. E. Stout, Santo Cristarella, Marco Quartuccio, Geert J. P. L. Kops, Marta De Ruijter-Villani

**Affiliations:** ^1^Department of Clinical Sciences, Faculty of Veterinary Medicine, Utrecht University, Utrecht, Netherlands; ^2^Department of Veterinary Sciences, Messina University, Messina, Italy; ^3^Oncode Institute, Hubrecht Institute–KNAW and University Medical Center Utrecht, Utrecht, Netherlands

**Keywords:** oocyte, aneuploidy, maternal aging, horse, spindle

## Abstract

Aneuploidy originating during meiosis in oocytes is the major cause of reduced fertility, implantation failure and miscarriage in women beyond their mid-thirties. Loss of chromosome cohesion, and defective microtubule dynamics and spindle assembly are, in turn, the major contributors to the error-prone nature of chromosome segregation in the oocytes of older women. However, the underlying molecular defects are not well understood. Altered function of MPS1 and AURKC have been shown to induce multipolar spindle phenotypes in murine oocytes and cancer cells, however, their role in reproductive aging associated oocyte aneuploidy is not known. Although age-related gamete and embryonic aneuploidy has been studied in female rodents, the horse may be a more appropriate animal model. Similar to women, aged mares suffer from reduced fertility and an increased incidence of oocyte aneuploidy. Moreover, mares show a long interval (decades) to reproductive senescence and, unlike rodents but similar to women, horse oocytes assemble the meiotic spindle in a slow and unstable manner, independent of microtubule organizing centers. In this study we found that oocytes from aged mares have lower expression of mRNA for *Mps1, Spc25* and *AurkC* than oocytes from young mares while gene expression for other meiosis regulators did not differ. To assess the ability of horse oocytes to correctly form a bipolar spindle, *in vitro* matured MII oocytes were allowed to re-form their spindle after nocodazole-induced microtubule depolymerization. To investigate the importance of MPS1 and AURKC function in spindle (re)assembly, various concentrations of a MPS1 inhibitor (MPS1i, Compound 5) or an AURK inhibitor (AURKi, ZM447439) were included after nocodazole washout. MII oocytes from aged mares showed a higher incidence of spindle abnormalities after exposure to MPS1i. In contrast, Aurora kinase inhibition severely impaired microtubule organization and spindle formation in all oocytes, irrespective of mare age. In conclusion, gene expression for the kinases *Mps1, Spc25*, and *AurkC* is reduced in oocytes from aged mares. Moreover, spindle (re)assembly in aged mares’ oocytes is more unstable when Mps1 is inhibited. Overall, this suggests that compromised Mps1 activity predisposes to meiotic spindle instability in aged mare oocytes. This spindle instability could predispose to chromosome segregation errors.

## Introduction

Aneuploidy originating during oocyte meiosis is the leading cause of pregnancy loss and infertility in women beyond their mid-thirties, with approximately 60% of their oocytes retaining the wrong number of chromosomes (Herbert et al., 2015; Gruhn et al., 2019, p. 1466–1469). Chromosomal analysis suggests that oocytes from older women fail to reliably protect centromeric cohesion and establish correct monopolar kinetochore–microtubule attachments (Herbert et al., 2015; Gruhn et al., 2019, p. 1466–1469; Zielinska et al., 2019, p. 3749–3765.e7). As women age, progressive weakening of sister chromatid cohesion in oocytes arrested in meiosis I prophase hinders the ability of sister kinetochores to form a single functional unit, and of sister chromatids to remain physically linked until the onset of anaphase of meiosis II (Shomper et al., 2014, p. 1171–1179; Herbert et al., 2015; Zielinska et al., 2015). Studies in mouse oocytes have demonstrated that cohesion loss is a major contributor to aneuploidy in aged females (Chiang et al., 2010, p. 1522–1528; Lister et al., 2010, p. 1511–1521; Burkhardt et al., 2016, p. 678–685). Moreover, loss of cohesins from centromeric regions induces broadening of the centromeric chromatin and fragmentation of the associated kinetochores (Zielinska et al., 2019, p. 3749–3765.e7); since fragmented kinetochores are more likely to interact with multiple microtubule bundles, they are prone to allowing attachment of microtubules emanating from opposite spindle poles (merotely) (Zielinska et al., 2019, p. 3749–3765.e7). Merotelically attached kinetochores/chromosomes are likely to lag behind at anaphase onset and give rise to chromosome segregation errors (Zielinska et al., 2019, p. 3749–3765.e7). Although cohesion loss is recognized as the major contributor to meiotic aneuploidy, other factors have been recognized to play an important role in the origin of meiotic segregation errors in human oocytes. Vulnerable crossover configurations, such as peri-centromeric, telomeric, or missing crossovers, appear to contribute to disruption of the “bivalent” structure (the association of two replicated homologous chromosomes) needed for accurate segregation during meiosis I (Shomper et al., 2014, p. 1171–1179; Herbert et al., 2015; Zielinska et al., 2015). Moreover, the intrinsic spindle instability typical of human oocytes, with up to the 80% of oocytes displaying transient multipolar spindles, has been proposed to predispose to chromosome segregation errors by promoting the establishment of incorrect merotelic mictotubule-kinetochore attachments (Holubcová et al., 2015, p. 572–573). A recent study on mice showed that oocyte spindle instability increases in aged individuals. However, altered microtubule dynamics were not attributable to age-related chromatin changes since transferring the nucleus of a young oocyte into the cytoplasm of an old one did not lead to a reduction of the incidence of multipolar spindle formation (Nakagawa and FitzHarris, 2017, p. 1040–1047). Although spindle instability is a well described phenomenon in human oocytes, the underlying molecular defects are not understood (Holubcová et al., 2015, p. 572–573; Nakagawa and FitzHarris, 2017, p. 1040–1047).

Mono Polar Spindle 1 (MPS1) kinase and Aurora kinase C (AURKC) are two of the master regulators of the meiotic divisions and known primarily for their roles in ensuring correct microtubule-kinetochore interaction and K-fiber assembly (Hached et al., 2011, p. 2261–2271); however they also localize to Microtubule Organizing Centers (MTOCs) and the spindle poles. MTOCs are the major microtubule nucleators during the process of murine oocyte spindle assembly and must cluster at the spindle poles to yield a stable bipolar spindle. A recent study in mice showed that the process of MTOC clustering at the oocyte spindle poles is very similar to centrosome clustering in cancer cells expressing supra-numerary centrosomes (Breuer et al., 2010, p. 1251–1260). Perturbation of MPS1 and AURK localization at the MTOCs and spindle poles has been shown to result in the inability to cluster MTOCs, resulting in multipolar spindle phenotypes in murine oocytes and cancer cells (Kwiatkowski et al., 2010, p. 359–368; Balboula and Schindler, 2014; Balboula et al., 2015, 2016, p. 3648–3660).

We therefore hypothesized that a reduction in MPS1 and AURKC kinase activity could play a role in reproductive aging-induced spindle instability and consequent aneuploidy in oocytes. The main obstacle to studying age-related compromise of oocyte spindle stability is the lack of an animal model in which spindle dynamics closely mimic those in human oocytes. Although rodents have been used in previous studies, their mechanism of meiotic spindle assembly and their chromosome conformation is different to that of human oocytes (Holubcová et al., 2015, p. 572–573; Mogessie et al., 2018). The similarities between women and mares with regard to the duration of oocyte prophase arrest (years to decades) and the time interval to reproductive senescence, suggest that the mare could represent a valuable “natural” model for studying the processes underlying reproductive aging. Both human and equine oocytes present meta- and submetacentric chromosomes, which are considered to present a greater structural challenge to the establishment of stable bipolar microtubule attachments than rodents’ telocentric chromosomes (Raudsepp and Chowdhary, 1999, p. 103–114; Holubcová et al., 2015, p. 572–573). Unlike rodents, human and horse oocytes assemble the meiotic spindle in a slow and unstable manner, independent of microtubule organizing centers (MTOCs) (Tremoleda et al., 2001, p. 260–269; Li et al., 2006, p. 661–667; Holubcová et al., 2015, p. 572–573). After nuclear envelope breakdown (NEBD), the microtubules start nucleating from the chromosomes, and γ-tubulin is only detectable on the minus ends of the microtubules as a diffuse staining at the spindle poles (Li et al., 2006, p. 661–667; Roeles and Tsiavaliaris, 2019). We recently showed that the age-related change in the incidence of aneuploidy in MII oocytes from mares (15% in young and 55% in old mares) is comparable to that reported for women (20% in women younger than 35 years; 60% in women older than 35 years) (Chiang et al., 2010, p. 1522–1528; Lister et al., 2010, p. 1511–1521; Fragouli et al., 2011, p. 286–295; Rizzo et al., 2020, p. 22220–22232). Moreover, the mechanics of mis-segregation appear to be analogous, since the majority of aneuploidies observed in aged mare oocytes were the result of premature separation of sister chromatids (Rizzo et al., 2020). The aim of the present study was to investigate the effects of advanced maternal age on MPS1 and AURKC gene expression, and examine whether function of these kinases plays an essential role in stabilizing spindle bipolarity in oocytes.

## Results

Six hundred and forty cumulus-oocytes-complexes (COCs) were collected from the ovaries of 118 mares (41 young and 77 old mares). Mare ages ranged between 2 and 14 years (mean ± SD: 8.9 ± 4.1 years) for the young group, and between 16 and 28 years (mean ± SD: 20.3 ± 3.7 years) for the old group.

For the gene expression study, 320 oocytes were divided into 8 groups on the basis of mare age (young vs. old), cumulus appearance before maturation (compact vs. expanded) and extrusion of the first polar body after maturation (MII and non-MII). For each of the 8 groups, 4 pools of 10 oocytes were used for mRNA extraction.

**FIGURE 1:**
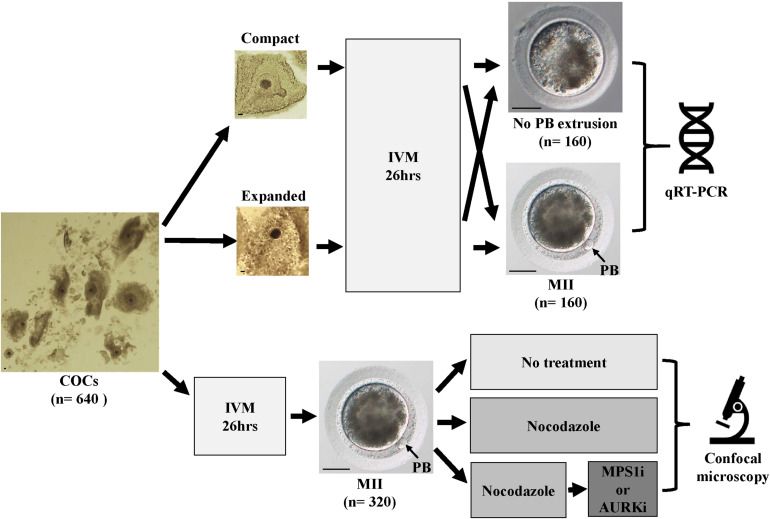
Schematic representation of the study design. Scale bars, 40 μm.

Three hundred and twenty oocytes that showed first polar body extrusion after *in vitro* maturation were randomly selected for examination of the effects of inhibitors and immunofluorescent analysis. Of the immune-stained oocytes, 68 (24 and 44 from young and old mares, respectively) had to be excluded due to inadequate immunofluorescent staining for tubulin or chromatin. A schematic representation of the study design, together with images of equine oocytes with or without a polar body, and an expanded or compact cumulus investment, are shown in Figure 1.

### Gene Expression for *MPS1* and *AURKC* Is Reduced in Oocytes From Aged Mares

**FIGURE 2:**
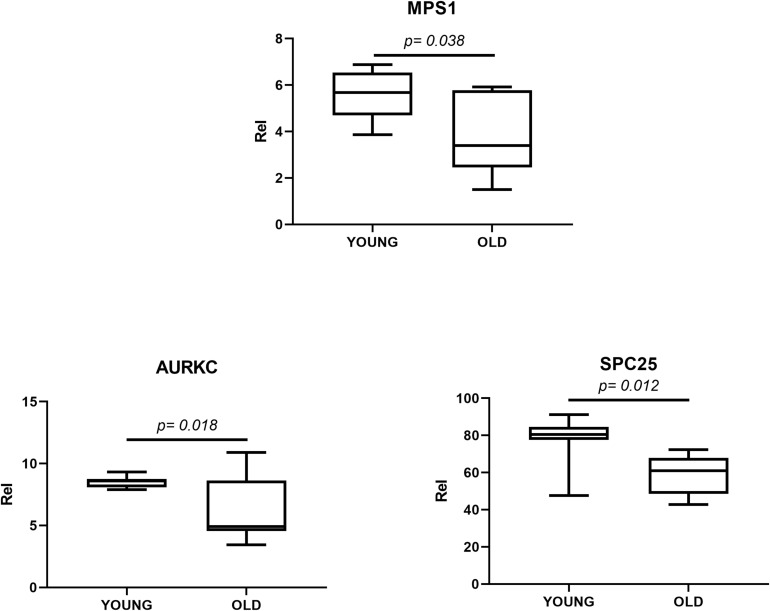
mRNA expression for *Mps1*, *Spc25* and *AurkC* in oocytes from young (≤14 years) and old (≥16 years) mares. The box plot shows the interquartile range, with the median value indicated by the horizontal line; whiskers show the range.

When we examined expression of a range of candidate regulators of chromosome segregation during meiosis, we found no effect of maturation stage (germinal vesicle vs. MII) or initial cumulus appearance (compact vs. expanded). However, we observed a significant reduction in gene expression for *MPS1*, *Spc25* and *AURKC* in oocytes from aged (≥16 years) compared to young (≤14 years) mares (Figure 2). By contrast, gene expression for *Mad2L2, Bub1, Bub3, Bub1B, Ndc80*, and *AURKB* was similar between the two age groups (Supplementary Figure 1).

To investigate the ability of oocytes to correctly form a bipolar spindle, *in vitro* matured MII oocytes were allowed to re-form a spindle after nocodazole-induced microtubule depolymerization. To further establish whether MPS1 and AURKC played important roles in spindle (re)assembly, increasing concentrations of a MPS1 inhibitor (MPS1i, Compound 5) or an AURK inhibitor (AURKi, ZM447439) were added to the post-nocodazole washout maturation medium. Morphology of MII spindles was classified as described by Baudoin and Cimini (2018, p. 215–227; Table 1).

**TABLE 1:** Diagrammatic representations and descriptions of all spindle abnormalities documented in the present study.

Spindle defects	Appearance	Description
Monopolar	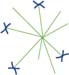	Presence of only one spindle pole.
Multipolar	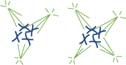	Presence of more than two spindle poles.
Bent	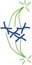	Spindle presenting a curved spindle axis instead of straight.
Absent	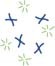	Failure of microtubules to organize in a spindle, absence of chromosome congression.

### Reduced Mps1 Function Induces Multipolar Spindle Formation in Oocytes From Aged Mares

**FIGURE 3:**
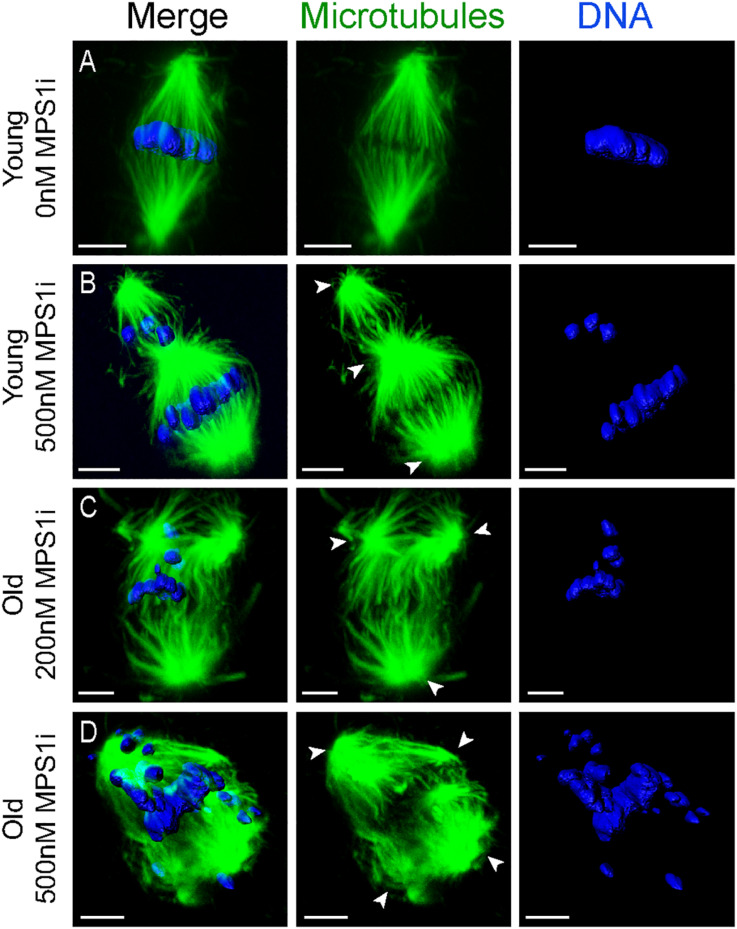
Representative images of spindles of horse MII oocytes treated with MPS1i after Nocodazole washout. Maximum intensity projections of confocal sections. Microtubules (alpha-tubulin, green); chromatin (Hoechst, blue). Scale bars, 5 μm. **(A)** Bipolar spindle from a young mare’s oocyte treated with 0 nM of MPS1i; **(B)** tripolar spindle from a young mare’s oocyte treated with 500 nM of MPS1i; **(C)** tripolar spindle from an old mare’s oocyte treated with 200 nM of MPS1i; **(D)** tetrapolar spindle from an old mare’s oocyte treated with 500 nM of MPS1i. Arrowheads indicate the spindle poles.

Oocytes from aged mares showed multipolar spindle formation after treatment with both tested concentrations of MPS1i, whereas oocytes from young mares only showed spindle abnormalities when exposed to the higher dose of inhibitor (Table 2). No abnormal spindles were observed in young mare oocytes that were not treated, were exposed to Nocodazole washout only or treated with 200 nM MPS1i after Nocodazole washout (0/60; Figure 3A). Only after treatment with 500 nM MPS1i following Nocodazole washout was a significant increase in the incidence of abnormal spindle formation observed (9/40) (23 vs. 0%, *P* = 0.021; Figure 3B). Similarly, no difference in the incidence of spindle abnormalities was observed in oocytes from aged mares not exposed to any treatment (5%, 1/19), or exposed to Nocodazole washout only (16%, 3/19). However, aged mares’ oocytes were susceptible to both concentrations of MPS1i, showing an increased incidence of tri- and tetrapolar spindles compared to the control group (7/21 [33%; *P* = 0.046] and 8/22 [36%; *P* = 0.024] for 200 and 500 nM MPS1i, respectively) (Figures 3C,D). The incidence of multipolar spindles was also significantly higher in aged compared to young mares’ oocytes treated with 200 nM of MPS1i (33% aged vs. 0% young; *P* = 0.01).

**TABLE 2:** Number, type and frequency of spindle abnormalities in MII oocytes from young (≤14 years) and old (≥16 years) mares, treated with MPS1i (0, 200, or 500 nM) or AURKi (0, 5, or 10 μM) after Nocodazole washout.

Maternal age (years)		≤14					≥16				
N. of abnormal spindles (%)		Monopolar	Multipolar	Bent	Absent	Total	Monopolar	Multipolar	Bent	Absent	Total
Treatment	Control	0	0	0	0	0/22 (0%)^a^	0	0	1	0	1/19 (5%)^a^
	0nM MPS1i/0μM AURKi	0	0	0	0	0/20 (0%)^a^	0	3	0	0	3/19 (15%)^ab^
	200nM MPS1i	0	0	0	0	0/18 (0%)^a^	0	6	1	0	7/21 (33%)^b^
	500nM MPS1i	0	7	2	0	9/40 (23%)^b^	0	8	0	0	8/22 (36%)^b^
	5μM AURKi	0	2	0	1	4/15 (27%)^b^	3	0	1	0	3/12 (25%)^ab^
	10μM AURKi	3	0	0	5	8/21 (38%)^b^	7	0	0	5	12/23 (52%)^b^

*Oocytes in the control groups were not treated with nocodazole nor inhibitors. Totals that share the same supe rscript do not differ significantly; P < 0.05.*

Of the oocytes with bipolar spindles, oocytes from aged mares showed a higher incidence of chromosome misalignment than those from young mares (Supplementary Table 1). This age-related difference was significant in every treatment group except the 200 nM MPS1i. Specifically, aged mare oocytes showed a total incidence of chromosome misalignment of 44% (8/18), 56% (9/16), 29% (4/14), and 71% (10/14) in the non-treated Control, Nocodazole, 200 and 500 nM MPS1i groups, respectively, compared to 5% (1/22; *P* = 0.006), 10% (2/20; *P* = 0.004), 22% (4/18; *P* = 0.7), and 23% (7/31; *P* = 0.002) for young mare oocytes (Supplementary Figure 2). No significant difference in the incidence of chromosome misalignment was observed between the different treatments within either age group.

### The Aurora Kinase Inhibitor AURKi Severely Impairs Microtubule Organization and Spindle Formation in Mare Oocytes

**FIGURE 4:**
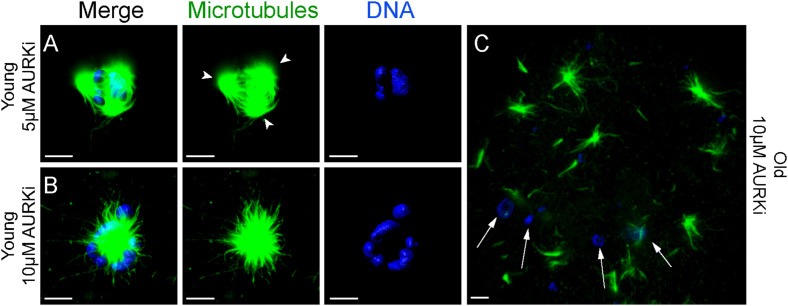
Representative images of spindles of horse MII oocytes treated with Aurki after Nocodazole washout. Maximum intensity projections of confocal sections. Microtubules (alpha-tubulin, green); chromatin (Hoechst, blue). Scale bars, 5 μm. **(A)** Tripolar spindle from a young mare’s oocyte treated with 5 μM of AURKi; arrowheads indicate the spindle poles; **(B)** monopolar spindle from a young mare’s oocyte treated with 10 μM of AURKi; **(C)** complete absence of spindle assembly and failure of chromosome congression from an old mare’s oocyte treated with 10 μM AURKi; white arrows indicate condensed chromatin dispersed within the cytosol.

Treatment with AURKi after Nocodazole washout increased the incidence of spindle abnormalities in oocytes from both young [27% (4/15; *P* = 0.021) and 38% (8/21; *P* = 0.001) for the 5 and 10 μM AURKi groups, respectively] and aged mares [52% (12/23) for the 10 μM AURKi group; *P* = 0.005], but not aged mare oocytes treated with 5 μM AURKi (25%, 3/12; *P* = 0.27) (Table 2). The most common abnormalities observed in oocytes treated with 5 μM AURKi were multipolar (in young mares oocytes; Figure 4A) or monopolar spindles (in aged mares oocytes; Figure 4B), while the most prominent abnormalities after treatment with 10 μM AURKi were monopolar spindles and the complete absence of spindle assembly and chromosome congression (Figure 4C). Oocytes treated with AURKi which did re-form a bipolar spindle, did not show an increased incidence of mis-aligned chromosomes compared to the control or Nocodazole treatment groups (Supplementary Table 1).

## Discussion

The results of the present study show that *in vitro* matured MII oocytes from aged mares have reduced mRNA expression for three meiotic regulators: *Mps1*, *AurkC*, and *Spc25*. We hypothesized that the reduced mRNA expression of *Mps1* and *AurkC* could result in a similar reduction in abundance of their respective proteins, leading to an impairment of their activity during the meiotic divisions. Although we attempted to assess Mps1, Spc25 and AurkC protein abundance in oocytes via immunofluorescence, the absence of adequately specific antibodies prevented us from being able to quantify their expression. Instead, to test the hypothesis that compromised Mps1 or AurkC activity may contribute to spindle instability in oocytes from aged mares, we evaluated the ability of oocytes from both young and old mares to re-assemble a metaphase II spindle after nocodazole-induced microtubule de-polymerization in the presence of different concentrations of an inhibitor for either Mps1 (MPS1i) or AURKC (AURKi); we were not able to obtain a validated inhibitor for Spc25. Whereas treatment with 500 nM MPS1i resulted in an increase in the incidence of tri- and tetrapolar spindles in oocytes from both young and old mares, treatment with 200 nM MPS1i induced multipolar spindles only in oocytes from old mares. The incidence of chromosome misalignment after MPS1i inhibition did not differ statistically when compared to the Nocodazole groups. Therefore, it seems that the inhibition of MPS1 in horse oocytes mainly affects bipolar spindle formation rather than chromosome alignment *per se*. The observation that oocytes from aged mares showed a higher frequency of misaligned chromosomes than oocytes from young mares, in all groups other than the 200 nM MPS1i treatment, is in accordance with previous observations in equine and human oocytes (Battaglia et al., 1996, p. 2217–2222; Rizzo et al., 2019, p. 252–257).

Mps1 has several functions during meiosis. Localization of Mps1 to the kinetochores is required for correct timing of prometaphase, correct AURKC localization and chromosome alignment, and to prevent precocious anaphase activation (Hached et al., 2011, p. 2261–2271). Mps1-depleted murine oocytes fail to correctly localize AURKC, suggesting that Mps1 is an important regulator of the chromosomal passenger complex (CPC; composed of AURKC, INCENP, survivin and borealin) in oocytes (Hached et al., 2011, p. 2261–2271; Nguyen et al., 2018, p. 3458–3468.e5). Moreover, Mps1 has been shown to localize at the spindle poles and Mps1-depleted oocytes show abnormal spindle formation; nevertheless, the exact function of Mps1 in microtubule clustering and spindle assembly in oocytes is still unknown (Hached et al., 2011, p. 2261–2271; Marquardt et al., 2016, p. 7828–7833). It has recently been shown that the CPC regulates both centrosome clustering in cancer cells with supernumerary centrosomes, and MTOC clustering in mouse oocytes (Leber et al., 2010; Balboula et al., 2016, p. 3648–3660). Mps1 disruption in cancer cells inhibits this clustering, leading to the formation of multipolar spindles (Kwiatkowski et al., 2010, p. 359–368). It is possible that, similar to cancer cells with supernumerary centrosomes, the clustering of the multiple acentrosomal spindle poles in oocytes lacking MTOCs is also regulated by Mps1 and CPC. It therefore seems reasonable to speculate that, as females age, impaired Mps1 function in their oocytes increases the risk of multipolar spindle formation.

In the present study, treatment with AURKi induced spindle abnormalities with a similar frequency in oocytes from both young and aged mares. The most frequent abnormalities observed were the formation of monopolar spindles and the complete absence of spindle assembly and chromosome congression. These findings are similar to what has previously been observed in Xenopus egg extracts and mouse oocytes treated with the same aurora kinase inhibitor (Wang et al., 2008, p. 1104–1111; Lane et al., 2010, p. 521–530). In Xenopus egg extracts, AurkB stabilizes spindle microtubules by inhibiting the activity of the catastrophe kinesin MCAK (Shao et al., 2012, p. 2672–2680; Ems-Mcclung et al., 2013, p. 2491–2499). Similarly, inhibition of AurkB/C in murine oocytes, by treatment with 5–10 μM AURKi, decreased MCAK phosphorylation and induced the formation of multipolar, monopolar and apolar spindles (Shuda et al., 2009, p. 1094–1105; Vogt et al., 2010, p. 665–684). We therefore propose that the spindle abnormalities observed in horse oocytes after Aurki treatment are a consequence of MCAK- dependent instability of the spindle microtubules. However since young and aged oocytes were similarly affected by this inhibition, this mechanism doesn’t seems to play an important role in reproductive aging associated oocyte aneuploidy.

## Conclusion

Oocytes from mares of advanced age show a reduced expression of genes encoding for three regulators of meiosis, *Mps1*, *AurkC*, and *Spc25*. Moreover, oocytes from aged mares show an increased sensitivity to Mps1 inhibition, but not to AURK inhibition, when compared to oocytes from young mares. We therefore propose that compromised Mps1 activity in oocytes from aged mares contributes to reproductive-aging induced spindle instability, which in turn might predispose to aneuploidy.

## Materials and Methods

### Oocyte Collection and *in vitro* Maturation

Ovaries were collected from slaughtered mares within 15 min of death, divided into two groups depending on the age of the mare (young, ≤14 years; old, ≥16 years) and transported to the laboratory within 4 h at 21–25°C as described previously (Rizzo et al., 2019, p. 252–257). Cumulus-Oocyte Complexes (COCs) were recovered from the ovaries by scraping the follicle wall and flushing out the dislodged COCs using embryo flushing medium (Euroflush, IMV Technologies, Leeuwarden, the Netherland) as previously described (Rizzo et al., 2019, p. 252–257). A dissecting microscope with 10–60× magnification was used for evaluating the recovered COCs. Only oocytes with at least one layer of intact cumulus cells were used for these studies; denuded oocytes were discarded. For the PCR experiment, COCs were further subdivided depending on cumulus morphology at recovery (Compact or Expanded). The oocytes were then washed in Hepes-buffered synthetic oviduct fluid (H-SOF; Avantea, Cremona, Italy) and subsequently matured *in vitro* for 26 h at 38.5°C in 5% CO_2_-in-air in a 50:50 mixture of Dulbecco’s minimal essential medium (DMEM) and Ham’s F12 (GIBCO BRL Life Technologies, Bleiswijk, The Netherlands) supplemented with 10% fetal calf serum (Sigma-Aldrich Chemical Co., St. Louis, Missouri, United States), 0.125 μg/mL epidermal growth factor (Peprotech Inc., Rocky Hill, New Jersey, United States), 0.1 IU/mL follicle-stimulating hormone, 0.6 mmol/L cysteine and 0.1 mmol/L cysteamine (Sigma-Aldrich Chemical Co.), 0.1% insulin, 0.1% transferrin and 0.1% sodium selenite (VWR International BV, Amsterdam, The Netherlands). After maturation, oocytes were denuded from the surrounding cumulus cells by gentle pipetting through a fine bore pipette, after a brief exposure to a 1 μg/mL solution of hyaluronidase (Sigma-Aldrich Chemical Co., St. Louis, Missouri, United States) in H-SOF. For the PCR experiments, oocytes were further divided into two groups depending on the presence or absence of the first polar body. For the inhibition experiments, only oocytes showing polar body extrusion were used.

### RNA Extraction, cDNA Synthesis and Quantitative Real Time-PCR (qRT-PCR)

Oocytes used for qRT-PCR were divided into 8 groups depending on the mare’s age (young or old), cumulus appearance before *in vitro* maturation (compact or expanded) and the presence or absence of a polar body after *in vitro* maturation (MII or non-MII). Oocytes were briefly washed in PBS (B. Braun, Hessen, Germany), and then transferred with a minimum amount of medium into pre-labeled cryovials, snap frozen in liquid nitrogen and stored at -80°C. Total RNA was extracted from pools of 10 oocytes (*n* = 4 per group) using the Invisorb^®^ Spin Cell RNA Mini Kit (Invitek, Berlin, Germany) according to the manufacturer’s instructions. Each pool of oocytes was lysed in 600 ml lysis buffer, and total RNA was eluted with 20 μl RNase-free water, after which RNA concentration and integrity were measured as described previously (de Ruijter-Villani et al., 2013, p. 979–989). The RNA was then treated with DNAse I (30 min at 37°C followed by 10 min at 65°C; 1 IU/mg of RNA; RNAse-Free DNase set, Qiagen Valencia, California, United States) and reverse transcribed into cDNA in a total volume of 20 μl containing 10 μl of sample using Superscript III (Invitrogen Corporation, Carlsbad, California, United States) as described previously (de Ruijter-Villani et al., 2013, p. 979–989). Minus RT blanks were prepared from 5 μl of sample under the same conditions, but in the absence of reverse transcriptase. Primer pairs (Table 3) for target genes (*Mad2, Bub1, Bub3, Bub1b, Mps1, Ndc80, Spc25, AurkB, AurkC*) and housekeeping genes (*Pgk1* and *Srp14*) were designed using PerlPrimer (Marshall, 2004, p. 2471–2472) and produced at Eurogentec (Seraing, Belgium). Primer specificity was tested by DNA sequencing (ABI PRISM 310 Genetic analyzer; Applied Bio-system, Foster City, California, United States). Real-time PCR was performed as described previously (de Ruijter-Villani et al., 2013, p. 979–989) in 15 μl of reaction mix that included 7.5 μl of IQ SYBR^®^ Green Supermix (BioRad, Veenendaal, Netherlands), 0.5 mM of primer, and 1 μl of cDNA, on an IQ5 Real-Time PCR detection System (BioRad). The cycle conditions were composed of an initial denaturation at 95°C for 3 min, followed by 40 amplification cycles consisting of 95°C for 3 min, the primer-specific annealing temperature (Table 3) for 30 s and 72°C for 45 s. A melting curve and standard curve were performed for each gene to verify product specificity and enable expression quantification. The stability of the housekeeping genes was determined using GeNorm (Vandesompele et al., 2002) and the geometric mean of the expression of SRP14 and PGK1 was used for normalizing the starting quantities of the target genes.

**TABLE 3:** Details of the primer pairs for target and housekeeping genes used in the present study.

Gene	Sequence	T_a_ (°C)	Amplicon size (bp)	GenBank Accession no.
MAD2L1	F: 5′-GCAGTTTGATATTGAGTGTGAC-3′ R: 5′-TCCTGATTCCTCCCATTTCTC-3′	58°	213	XM_001503199.3
BUB1	F: 5′-GACTCAAATACGGAACAAAGG-3′ R: 5′-TCTGTCTTCATTTACCCACTG-3′	60°	209	XM_005599825.1
BUB3	F: 5′-GAAGTACGCCTTCAAGTGTC-3′ R: 5′-TTTACGAATCCATCAGAACCAC-3′	62°	125	XM_005602172.1
BUB1B	F: 5′-GCAGATATGATATTTCAGGAAGGG-3′ R: 5′-CTGGTTTGAAGCCTTGAGAG-3′	58°	251	XM_005603154.1
MPS	F: 5′-GGTTCAAGTAAGGTATTTCAGG-3′ R: 5′-ATTTCCACACTCCATTACCA-3′	58°	215	XM_001499324.3
NDC80	F: 5′-AGTTGAATATAAGCAGACCCAC-3′ R: 5′-GCTTGTAGGGACTTCATGGA-3′	62°	231	XM_001492535.3
SPC25	F: 5′-GCAGACTTGTATAAAGATCGAC-3′ R: 5′-CTATCTGACACTTCATAGTCCC-3′	60°	157	XM_005601567.1
AURKB	F: 5′-GAAGAAGAGCCATTTCATCGT-3′ R: 5′-ACTCCAGAATCAAGTAGATCCT-3′	62°	178	XM_001504814.3
AURKC	F: 5′-GCATCTACAACACCCAAATATCC-3′ R: 5′-GTTCTCGGGCTTAATATCCCT-3′	60°	225	XM_005596580.1
PGK1	F: 5′-CTGTGGGTGTATTTGAATGG-3′ R: 5′-GACTTTATCCTCCGTGTTCC-3′	54°	151	XM_005614287.1
SRP14	F: 5′-CTGAAGAAGTATGACGGTCG-3′ R: 5′-CCATCAGTAGCTCTCAACAG-3′	55°	101	XM_001503583.3

### Nocodazole, MPS1i and AURKi Treatment of Oocytes

The function of the two kinases Mps1 and AurkC was investigated using two inhibitory drugs, namely Mps1i or compound 5 (kindly supplied by Prof. Geert Kops Hubrecht Institute, Utrecht, the Netherlands) (Kwiatkowski et al., 2010, p. 359–368) and AURKi or ZM447439 (CAS 331771, Sigma-Aldrich Chemical Co., St. Louis, Missouri, United States). In order to evaluate the function of Mps1 and AurkC on spindle assembly, mature oocytes displaying a polar body were first incubated for 10 min at 38°C with 5% CO_2_ in IVM medium containing 20 μM Nocodazole (M1404, Sigma-Aldrich Chemical Co., St. Louis, Missouri, United States) to depolymerize the MII-spindle. Following Nocodazole washout, the oocytes were left to re-form their spindle for 120 min in IVM medium with different concentrations of either MPS1i (0, 200, or 500 nM) or AURKi (0, 5, or 10 μM). The oocytes in the control group were not treated with nocodazole or inhibitors.

### Oocyte Fixation

Before fixing, all oocytes were exposed to cold shock on ice for 9 min to depolymerize unstable microtubules. The oocytes were then treated with a glycerol-based microtubule-stabilizing solution (Medium M) for 1 h at 37°C (Simerly and Schatten, 1993), (25% (v/v) glycerol, 50 mM KCl, 0.5 mM MgCl_2_, 0.1 mM EDTA, 0.1 mM EGTA, 1 mM 2-mercaptoethanol, 50 mM imidazole, 4% Triton-X-100, and 25 mM phenylmethylsulphonylfuoride; pH 6.7: all from Sigma-Aldrich Chemical Co.), and subsequently fixed with a 2% solution of paraformaldehyde (Electron Microscopy Sciences, Hatfield, Pennsylvania, United States) in PBS.

### Immunostaining, Confocal Imaging and 3-Dimensional Image Analysis

Fixed oocytes were immunostained for α-tubulin and chromatin as described previously (Rizzo et al., 2019, p. 252–257). Shortly, oocytes were washed in PBS with 3 mg/ml PVP (Sigma-Aldrich Chemical Co.) (PBS-PVP) three times for 5 min before being incubated over-night in PBS containing 1:250 mouse monoclonal anti-α-tubulin antibody (T5168, Sigma-Aldrich Chemical Co.) at 4°C. Oocytes where then washed twice for 5 min in a 0.1% solution of BSA (Sigma-Aldrich Chemical Co.) in PBS and incubated for 1 h at Room Temperature (RT) in blocking solution containing 0.1 M glycine, 1% goat serum, 0.01% Triton X-100, 0.5% BSA, and 0.02% sodium azide (all from Sigma-Aldrich Chemical Co.), followed by incubation in the dark at 37°C in PBS containing 0.5% Triton X-100, 0.5% BSA and a 1:100 Goat Anti-Mouse Alexa Fluor^®^ 488 antibody (A11029, Invitrogen Corporation, Carlsbad, California, United States). After two washing steps of 5 min in PBS containing 0.1% BSA and 0.1% Triton X-100, and two washing steps of 5 min in PBS alone, the oocytes were incubated in the dark for 30 min at RT in PBS-PVP containing 5 μg/ml of Hoechst (Hoechst 33342, Sigma-Aldrich Chemical Co.). After a brief wash in PBS-PVP, the oocytes were mounted on glass slides (SuperfrostPlus; Menzel, Braunschweig, Germany) with Vectashield (Vector Laboratories, Burlingame, California, United States) to avoid photobleaching. Image acquisition was performed using a confocal laser scanning microscope (Leica TCS-SPE-II; Leica Microsystems, Wetzlar, Germany) equipped with a 63× objective. Hoechst 33342 was stimulated with a 405 nm laser and the emission was detected between 414 and 466 nm (blue channel), Alexa Fluor 488 was separately stimulated with a 488 nm laser and emission was detected in the 511–577 nm range (green channel). Metaphase II spindles were identified, and sequential sections were taken throughout the whole spindle at 0.42-μm intervals (z-step size). Image-acquisition and analysis of spindle morphology and chromosome alignment were performed using 3D analysis software (Imaris 8.1; Bitplane AG, Zurich, Switzerland) as described previously by Rizzo et al. (2019, p. 252–257). Gross morphology of MII spindles were classified as described by Baudoin and Cimini (2018, p. 215–227; Table 1). Chromosome misalignment was classified as absent when all chromosomes were on the metaphase plate, mild if up to 5 chromosomes were displaced from the spindle equator and severe if more than 5 chromosomes were displaced from the spindle equator, as described previously (Rizzo et al., 2019, p. 252–257).

### Statistical Analysis

QRT-PCR data were analyzed using SPSS 16.0 for Windows (SPSS Inc., Chicago, IL). To obtain continuous normally distributed data sets, the relative starting quantities were subjected to natural logarithmic transformation. Data were analyzed using a two-way between-groups ANOVA, followed by a *post hoc* Tukey test. When a significant effect of the interaction was found, the analysis of simple effects was conducted by running separate one-way ANOVAs.

The incidence of spindle abnormalities and chromosome misalignment between age and treatment groups were compared by Fisher’s exact and Cochran-Mantel-Haenszel tests, using a Chi-squared analysis performed using IBM SPSS Statistics for Windows (Version 24.0, Armonk, New Your, United States). Results were considered statistically significant when *P* ≤ 0.05.

## Data Availability Statement

The raw data supporting the conclusions of this article will be made available by the authors, without undue reservation.

## Author Contributions

MD and MR-V conceived the project, further designed and conducted the experiments and wrote the original draft of the manuscript. MD performed the formal analysis. TS and GK contributed to conception of the work and edited the manuscript. SC and MQ edited the manuscript. All authors contributed to the interpretation of the data and read and approved the final manuscript.

## Conflict of Interest

The authors declare that the research was conducted in the absence of any commercial or financial relationships that could be construed as a potential conflict of interest.

## Supplementary Material

Supplementary Figure 1 mRNA expression for *Mad2L2, Bub1, Bub3, Bub1B, Ndc80*, and *AURKB* in oocytes from young (≤14 years) and old (≥16 years) mares. The box plot shows the interquartile range, with the median value indicated by the horizontal line; whiskers show the range.

Supplementary Figure 2 Representative images of spindles of MII oocytes showing chromosome misalignment. Maximum intensity projections of confocal sections. Microtubules (alpha-tubulin, green); chromatin (Hoechst, blue). Scale bars, 5 μm. **(A)** bipolar spindle with all chromosomes on the metaphase plate from a young mare’s oocyte treated with 0 μM of MPS1i; **(B)** bipolar spindle with mild chromosome misalignment from an old mare’s oocyte treated with 0 μM of MPS1i; **(C)** bipolar spindle with mild chromosome misalignment from an old mare’s oocyte treated with 0 μM of MPS1i; **(D)** bipolar spindle with severe chromosome misalignment from an old mare’s oocyte treated with 500 μM of MPS1i.

Supplementary Table 1 Number, type and frequency of mild and severe chromosome misalignment in bipolar spindles of MII oocytes from young (≤14 years) and old (≥16 years) mares, treated with MPS1i (0, 200, or 500 nM) or AURKi (0, 5, or 10 μM) after Nocodazole washout. Oocytes in the control groups were not treated with nocodazole or an inhibitor. Totals that share the same superscript do not differ significantly; *P* < 0.05.
