# Supplementary figures and images for: Compromised MPS1 Activity Induces Multipolar Spindle Formation in Oocytes From Aged Mares: Establishing the Horse as a Natural Animal Model to Study Age-Induced Oocyte Meiotic Spindle Instability
Source: Front Cell Dev Biol. 2021 May 6;9:657366. doi: 10.3389/fcell.2021.657366 (PMC8136435; doi:10.3389/fcell.2021.657366)

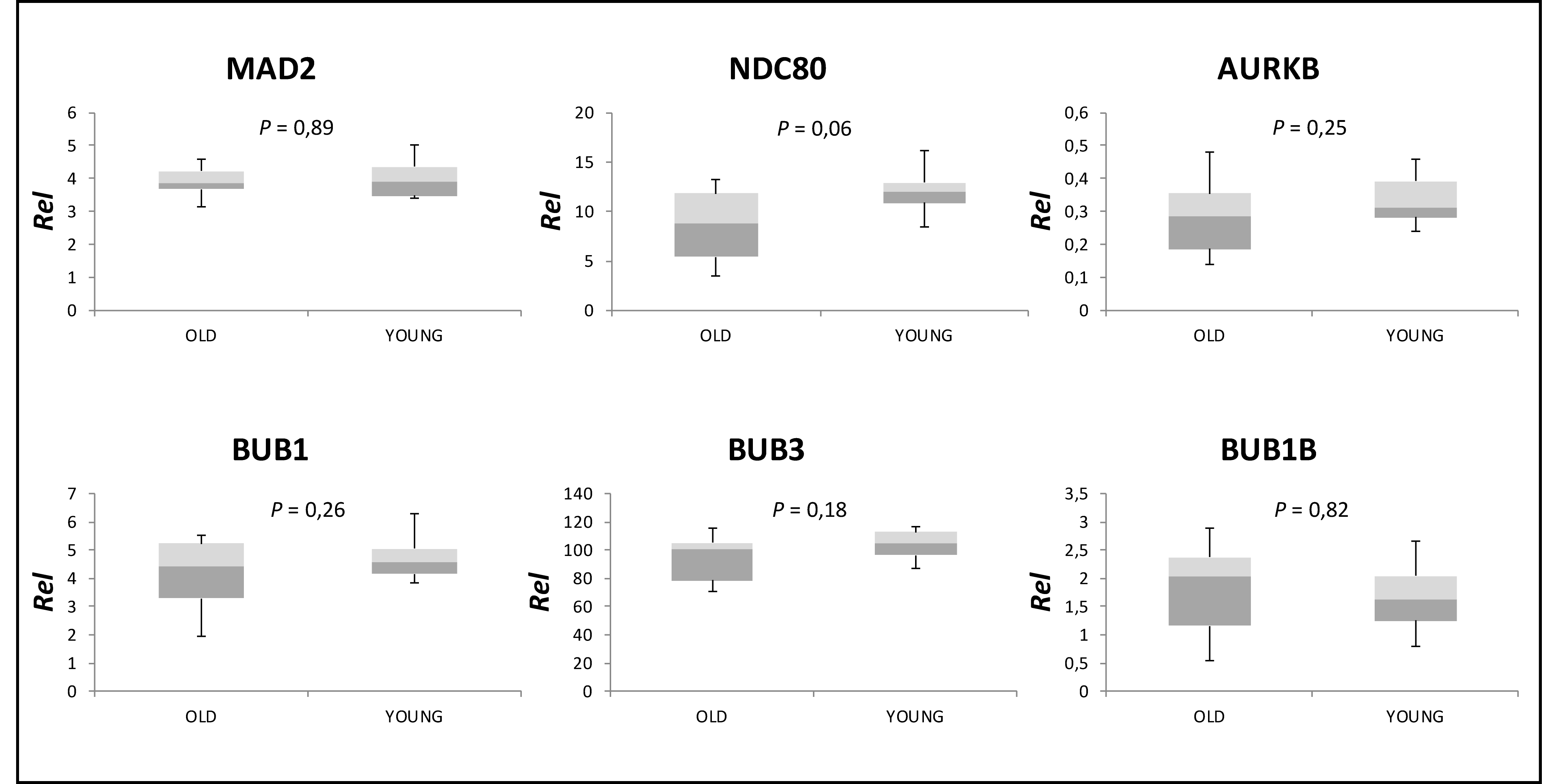

Supplement: Supplementary Figure 1 — mRNA expression for Mad2L2, Bub1, Bub3, Bub1B, Ndc80, and AURKB in oocytes from young (≤14 years) and old (≥16 years) mares. The box plot shows the interquartile range, with the median value indicated by the horizontal line; whiskers show the range. [file Image_1.TIF]

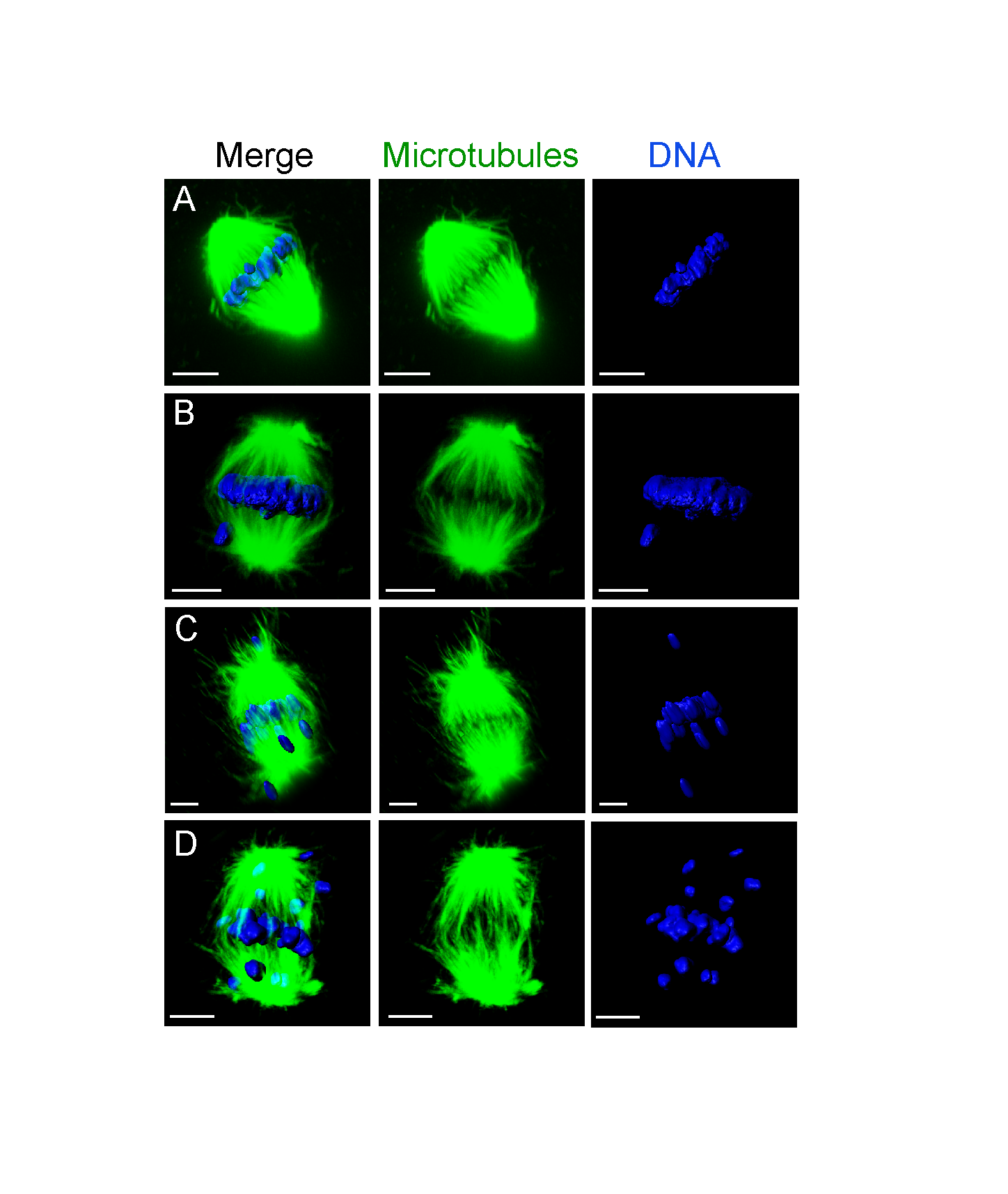

Supplement: Supplementary Figure 2 — Representative images of spindles of MII oocytes showing chromosome misalignment. Maximum intensity projections of confocal sections. Microtubules (alpha-tubulin, green); chromatin (Hoechst, blue). Scale bars, 5 μm. (A) bipolar spindle with all chromosomes on the metaphase plate from a young mare’s oocyte treated with 0 μM of MPS1i; (B) bipolar spindle with mild chromosome misalignment from an old mare’s oocyte treated with 0 μM of MPS1i; (C) bipolar spindle with mild chromosome misalignment from an old mare’s oocyte treated with 0 μM of MPS1i; (D) bipolar spindle with severe chromosome misalignment from an old mare’s oocyte treated with 500 μM of MPS1i. [file Image_2.tif]
