# Supplementary material for: Compromised MPS1 Activity Induces Multipolar Spindle Formation in Oocytes From Aged Mares: Establishing the Horse as a Natural Animal Model to Study Age-Induced Oocyte Meiotic Spindle Instability
Source: Front Cell Dev Biol. 2021 May 6;9:657366. doi: 10.3389/fcell.2021.657366 (PMC8136435; doi:10.3389/fcell.2021.657366)
Supplement: Supplementary Table 1 — Number, type and frequency of mild and severe chromosome misalignment in bipolar spindles of MII oocytes from young (≤14 years) and old (≥16 years) mares, treated with MPS1i (0, 200, or 500 nM) or AURKi (0, 5, or 10 μM) after Nocodazole washout. Oocytes in the control groups were not treated with nocodazole or an inhibitor. Totals that share the same superscript do not differ significantly; P < 0.05. [file Table_1.pdf]

Maternal age (years)		≤14			≥16		
**N. of oocytes with bipolar spindles showing misaligned chromosomes (%)**		Mild (≤ 5 chrom.)	Severe (>5 chrom.)	Total	Mild (≤ 5 chrom.)	Severe (> 5 chrom.)	Total
**Treatment**	Control	1	0	1/22 (5%)[a]	8	0	8/18 (44%)[b]
	0nM MPS1i/0μM AURKi	3	0	0/20 (0%)[a]	5	0	9/16 (56%)[b]
	200nM MPS1i	0	0	0/18 (0%)[a]	0	6	4/14 (28%)[b]
	500nM MPS1i	0	7	9/40 (23%)[a]	0	8	10/14 (71%)[b]
	5μM AURKi	1	0	1/11 (9%)[ab]	4	0	4/9 (44%)[ab]
	10μM AURKi	3	0	8/21 (38%)[ab]	0	0	0/11 (52%)[ab]
